# Injury pattern, outcome and characteristics of severely injured pedestrian

**DOI:** 10.1186/s13049-015-0137-8

**Published:** 2015-08-05

**Authors:** Georg Reith, Rolf Lefering, Arasch Wafaisade, Kai O. Hensel, Thomas Paffrath, Bertil Bouillon, Christian Probst, TraumaRegister DGU

**Affiliations:** Department of Trauma and Orthopedic Surgery, Cologne-Merheim Medical Center (CMMC), Witten/Herdecke University, Cologne, Germany; Institute for Research in Operative Medicine, IFOM, Witten/Herdecke University, Cologne, Germany; ZBAF, Center for Biomedical Education and Research, Witten/Herdecke University, Witten, Germany; Klinik für Unfallchirurgie, Orthopädie und Sporttraumatologie, Lehrstuhl der Universität Witten/Herdecke, Klinikum Köln-Merheim der Kliniken der Stadt Köln gGmbH, Ostmerheimerstr. 200, Köln, Germany

## Abstract

**Background:**

Pedestrians who are involved in motor vehicle collisions present with a unique trauma situation. The aim of this study was to demonstrate the specific clinical characteristics of this patient population in comparison to injured motor vehicle occupants in the medical emergency setting.

**Methods:**

A total of 4435 pedestrian traffic collision victims admitted to hospitals participating at TraumaRegister DGU® between 2002 and 2012 (primary admission, Injury Severity Score, ISS ≥ 9; age ≥ 2 years) was assessed and compared to 16,042 severely injured motor vehicle occupants. Analyses included features such as demographic distribution, injury patterns, treatment course, subsequent complications and overall clinical outcome.

**Results:**

Severely injured pedestrians more commonly were female (42 % vs. 34 % of motor vehicle occupants) and children below 16 years (12 % vs. 2 %) or seniors above 60 years of age (39 % vs. 17 %). Pedestrians were injured more severely (ISS: 26 vs. 25; NISS 32 vs. 30) with higher rates of head injuries (64 % vs. 47 %), pelvic injuries (32 % vs. 23 %) and lower extremity injuries (52 % vs. 43 %). Accordingly, pedestrians more commonly presented with Glasgow Coma Scale <9 (36 % vs. 28 %) and a systolic blood pressure below 90 mmHg (18 % vs. 13 %) accumulating in a worse prognosis (RISC-Score 24 % vs. 15 %) despite of a shorter on-scene treatment time (26 min vs. 38 min) and a shorter period from the collision until hospital admission (61 min vs. 78 min). Finally, pedestrians showed a higher mortality (22 % vs. 12 %).

**Conclusion:**

Severely injured pedestrians represent a challenging patient population with unique injury patterns and high subsequent mortality. Emergency team members should be sensitized to the trigger term “pedestrian” in order to improve the initial emergency management and thus the overall clinical outcome.

## Background

Worldwide, road traffic injuries are a leading cause of death with more than 1.2 million fatalities each year [1]. In Germany 15 % of road traffic collisions are pedestrian motor vehicle collisions (PMVCs) [2]. As the force of a road traffic collision usually hits the unprotected body of an involved pedestrian, severe injuries occur following unique mechanisms and kinematics [3, 4]. PMVCs comprise three major physical impacts: the bumper, the hood and windscreen and the ground impact [4, 5]. Based upon this, specific injury patterns in PMVCs could be described. In 1965, Farley introduced the term “fatal triad” as a combination out of injuries of the lower extremities, the pelvis and the head [6]. In 1971, Waddel refined this theory and adapted Farley’s “fatal triad” into a combination of injuries of head, pelvis/hip, and knee region and even declared a scenario of a severely injured pedestrian without this specific pattern of injuries as unimaginable [7]. Over the past 40 years, contradicting literature was published in various countries regarding this topic. While several authors aimed to object the theories of Waddel and Farley, the significance of most studies is limited by methodological aspects such as small study populations [8–11]. In a number of studies no specific injury combination could be identified but lower extremity musculoskeletal injuries could be labeled as the most common injuries in victims of PMVCs [5, 12–14].

Beside a specific injury pattern several authors have analyzed the demographic distribution in PMVCs [9, 13, 15]. These analyses revealed in the industrialized world older individuals as an important subset of pedestrian traffic collision victims that was often associated with a poor clinical outcome.

The objective of this study was to describe and characterize injured pedestrians as a vulnerable subpopulation of severely injured accident victims with regard to epidemiology, injury patterns, treatments as well as clinical outcome in comparison to the injured motor vehicle occupants in order to tailor a medical treatment to the specific needs of this patient population.

## Methods

All data was provided by TraumaRegister DGU® (TR-DGU), a large multi-centre database for anonymous and standardized documentation of severely injured patients that was initiated by the German Trauma Society in 1993. Standardized documentation includes detailed information on demographics, injury patterns, comorbidities, pre- and in-hospital medical management including intensive care unit treatment, relevant laboratory findings and finally the medical outcome of each individual. Standardized scoring systems utilized are the Injury Severity Score (ISS) [16] and the Abbreviated Injury Scale (AIS) (2005 version) [17]. The TraumaRegister DGU® is a voluntary registry, and participation is free of charge. As a compulsory tool for quality assessment, no informed consent is necessary for data collection. However, hospitals agree to scientific evaluation of contributed data that has been identified. Prior to dataset analysis, scientists have to apply to use data in written form, which explains the key question and scientific background of the project. After approval by the institutional review board, the study will be registered and results as well as its publication will be reviewed internally and recorded [18]. Participation in TraumaRegister-DGU® and analysis of data are approved by the participants’ institutional ethical review boards. Institutional ethical review board agreement documents were not administered by TraumaRegister DGU®.

Documented data is primarily out of hospitals located in Germany (90 %), with a growing number of participating hospitals in other countries, such as Austria, Belgium, China, Finland, Luxembourg, Slovenia, Switzerland, the Netherlands, and the United Arab Emirates. Currently, approximately 25,000 cases are entered into the databank by more than 600 hospitals every year.

In this study, we analyzed a dataset documented in the TraumaRegister DGU® of the years 2002 to 2012 (primary admission, Injury Severity Score (ISS) ≥ 9 plus intensive care treatment; age ≥ 2 years). We carried out a descriptive characterization of injured pedestrians (*n* = 4435) and compared the findings with a control group of motor vehicle occupants involved in road traffic collisions (*n* = 16,042). The term motor vehicle includes in our analyses passenger cars, sport utility wagons, buses and trucks. Motorcyclists were not integrated in our study. Inclusion criteria was an ISS ≥ 9 plus the treatment on an intensive care unit. Any injury scored AIS 2 or higher was analyzed. The ISS score of 9 or higher was chosen to include patients with severe lower extremity trauma but without severe trauma to the thorax or abdomen. Shock was defined in the TR-DGU by a systolic blood pressure below 90mmHG.

The prognosis was estimated based on the Revised Injury Severity Classification Score (RISC-Score) [19], utilizing the following parameters: age, New Injury Severity Score, head injury, severe pelvic injury, Glasgow Coma Scale, pre-hospital cardiac arrest, partial thromboplastin time (PTT), base excess (BE), hemoglobin (Hb), pre-hospital systolic blood pressure, and mass transfusion.

All statistical analyses have been performed with the commercially available computer software SPSS statistical software (IBM Inc., Armonk NY, USA). Categorical variables were presented as percentages. Continuous variables were presented as mean +/− standard deviation (SD). Given the large sample size of this descriptive analysis, the use of formal statistically testing was deliberately avoided since even minor differences would turn out to be statistical relevant. With a sample size of 4435 the effect size (the range of a 95 % confidence interval) is approximately +/− 1.5 % for categorical variables, and +/− 0.04*SD for continuous variables. For the control group with 16,042 cases the differences are even smaller. The present study is in accordance to the publication guidelines of TraumaRegister DGU® and registered as TR-DGU project ID 2012–058.

## Results

There are several important differences between the study group of severely injured pedestrians (*n* = 4435) and the control group of injured motor vehicle occupants (*n* = 16,042). The rate of women in the group of injured pedestrians was 42 % compared to 34 % in motor vehicle occupants. On average, pedestrians had a mean age approximately 10 years older than the control group (49.1 +/− 25). Children below the age of 16 represented 12 % of the severely injured pedestrians but only 2 % of motor vehicle occupants. 39 % of the pedestrians were older than 60 years (vs. 17 % in the control group) (Table 1).

**Table 1:** Demographic features, pre-hospital data and clinical characteristics of pedestrians and motor vehicle occupants involved in road traffic collisions

		Pedestrians (*n* = 4435)	Motor vehicle occupants (*n* = 16,042)
General data	Women (%)	42	34
	Mean age (years)	49.1 (+/− 25.0)	38.7 (+/− 19.4)
	Mortality (%)	21,7	12,3
Regional injury severity	Head trauma (AIS ≥3) (%)	56.6	40.7
	Chest trauma (AIS ≥3) (%)	42.8	60.1
	Abdominal trauma (AIS ≥3) (%)	12.0	20.8
	Extremities (including pelvis, AIS ≥3) (%)	44.8	40.8
Pre-hospital setting	Collision scene to hospital time (min)	61 (+/− 35)	78 (+/− 36)
	On-scene time (min)	25 (+/− 14)	37 (+/− 21)
In hospital treatment and clinical course	Time in the emergency room (min)	70 (+/− 44)	73 (+/− 45)
	Blood transfusion rate (%)	26.2	24.2
	Surgery (%)	81.2	85.3
	Time of ventilation (days)	6.4 (+/− 11)	6.2 (+/− 10.6)
	ICU length of stay (days)	10.4 (+/−14.0)	10.2 (+/−12.6)
	Hospital length of stay (days)	23.4 (+/−24.0)	24.0 (+/− 24.0)

*AIS*, abbreviated injury scale

**Fig. 1:**
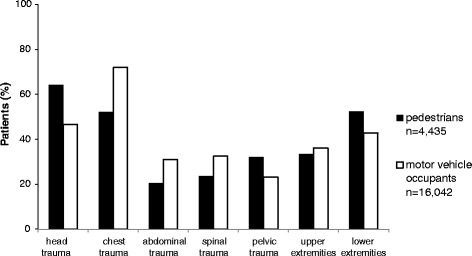
Comparison of injured body regions (AIS ≥ 2) in pedestrians and motor vehicle occupants injured in road traffic collisions

The differences in injured body regions are shown in Fig. 1. The different injury patterns are illustrated in Table 2. The severity of the different AIS regions in both study groups is given in Table 1.

**Table 2:** Injury patterns in pedestrians and motor vehicle occupants

Injury pattern			Pedestrians	Motor vehicle occupants
Head	Chest	Lower extremity	16.3 %	11.9 %
Head	Chest	Upper extremity	13.4 %	12.5 %
Chest	Upper extremity	Lower extremity	13.0 %	13.0 %
Chest	Pelvic	Lower extremity	11.4 %	8.8 %
Head	Upper extremity	Lower extremity	11.3 %	6.8 %
Head	Chest	Pelvis	10.7 %	7.9 %
Head	Pelvis	Lower extremity	10.6 %	4.6 %

**Fig. 2:**
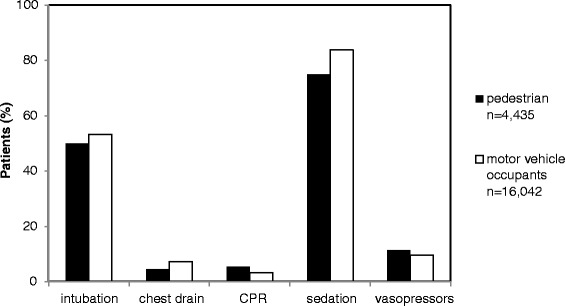
Distribution of pre-hospital interventions in pedestrians and motor vehicle occupants injured in road traffic collisions

Pedestrians had a mortality almost twice as high as motor vehicle occupants (21.7 % vs. 12.3 %). Furthermore, the pedestrian group had a higher mean RISC-Score (23.8 % vs. 14.5 %) and was overall injured slightly more severely (ISS 26.2 vs. 25.4 points; NISS 31.9 vs. 30.0 points). Interestingly, this negatively correlates both with the on-scene treatment time, which was approximately 12 min faster in the pedestrian group (26 vs. 38 min) as well as with the entire emergency transport operating time (61 vs. 78 min). On average the emergency physician needed 4 min less to reach the collision scene of a PMVC (mean 15 min) and the on-scene treatment time in PMVCs was 12 min shorter (mean 25 min) compared to the control group of motor vehicle occupants (Table 1). On average, during pre-hospital emergency care pedestrians were administered significantly less intravenous fluids compared to the control group (1086 +/− 820 vs. 1408 +/−952 ml) (Fig. 2).

The other pre-hospital interventions are presented in Fig. 2.

Injured pedestrians more often showed an initial Glasgow Coma Scale below 9 (35.5 % vs. 27.7 % in motor vehicle occupants) and a lower rate of pre-hospital intubations (50.0 % vs. 53.3 %). Pedestrians reached the hospital emergency department more often in severe shock (18.0 % vs. 13.6 %). Moreover, pedestrians had a slightly lower mean Hb level (11.4 +/− 2.7 vs. 11.7 +/−2.9 mg/l), a higher PTT level (36 +/−22 vs. 34.7 +/−20 s) and a lower arterial base excess level (−3.5 vs.–3.1) upon arrival at the treating hospital. In addition, pedestrians died more often during the first 24 h after hospital admission (13.2 % vs. 7.0 %). In-hospital treatment and length of stay is illustrated in Table 1.

## Discussion

The main findings of our study were, firstly, that compared to motor vehicle occupants more women, children and elderly citizens were involved in PMVCs. Secondly, in PMVCs victims head, pelvis and lower extremities were more commonly and more severely injured than in the motor vehicle occupant group. Concerning injury combination, the combination of head, chest and the lower extremities was seen most frequently in PMVCs. Thirdly, injured pedestrians showed a higher mortality compared to motor vehicle occupants in spite of a shorter rescue time and nearly similar ISS.

In our study a higher rate of elderly patients was involved in a collision as pedestrians than as motor vehicle occupants. These findings are in accordance with recent data from several authors. A commonly accepted explanation is, that elderly more likely participate in road traffic as pedestrians and more often sustain a collision because of their limited physiological capacities [14, 15, 20]. Furthermore, when involved in a collision the risk for elderly people to suffer severe injuries is significantly higher compared to younger individuals experiencing a comparable accident mechanism [9, 15]. Strikingly, women were overrepresented in the pedestrian group when compared to motor vehicle occupants. Barely any evidence is known regarding this gender distribution in collision trauma patients. One possible explanation might be the higher life expectancy of women in combination with the above mentioned finding [21]. Furthermore, gender differences regarding access to motor vehicles still occur in the western world. Although few data is known that supports these hypotheses, in Germany men above the age of 65 are known to outweigh women of the same age in terms of possession of a driving license. Concomitantly, no relevant gender differences are known in younger driving license holders [22]. The higher rate of children in PMVCs is not surprising and most probably a consequence of their unpredictable behavior as road traffic participants [23]. Besides that, studies have proven children to have longer reaction times and less established locomotory capacities, which additionally make them more prone to be victims in PMVCs [24, 25].

Our results reveal that both injury patterns and severity differ notably between victims of PMVCs and motor vehicle occupants. This is in line with findings of several other studies [26–29]. In PMVCs the force of the collision usually hits the unprotected body of a pedestrian. This lack of physical protection against the impact makes pedestrians - like bicyclists and motorbike drivers - an especially vulnerable road user group. Importantly, motor vehicle occupants in our study showed a higher rate of injuries to the torso while pedestrians more commonly were injured at peripheral body regions such as head and extremities. This is in accordance to earlier publications [5, 13, 14, 30–32]. The high rate of severe head injuries is surely one reason for the low initial GCS (35 % below 9 in the pedestrian group) and jointly responsible for the high rate of pre hospital intubations.

This general PMVC injury pattern is supported by the classic theory of PMVC kinematics [4]. However, more recent investigations demonstrated significant inter-individual variations in common PMVC injury combinations that are influenced by several factors (e.g., vehicle type, body region of first impact, main impact direction etc.) [8, 12, 9–11, 30]. Our data confirms this conclusion. We were unable to identify a classic pedestrians’ “fatal triad” of injuries as described by Farley and Waddell for severely injured PMVC victims [6, 7, 12]. Yet, there may remain differences in terms of inclusion criteria such as injury severity. Furthermore, investigations by Farley and Waddell did not utilize the AIS score as a measure for coherence of injury patterns and severity. For a comprehensive picture of the differences in injury type and severity, not only the pedestrian involved but also the external circumstances such as the type of vehicle, raod and traffic circumstances have to be taken into account [3, 5, 33–35]. All together it has to be stated, that the “fatal triad” theory has nowadays less relevance.

Strikingly, in this study pedestrians had a significantly higher mortality than motor vehicle occupants (21.7 % vs. 12.3 %) despite shorter on-scene treatment and entire emergency transport operating times. This is in contrast to the commonly accepted paradigm, that in trauma care of severely injured patients, time is a crucial parameter [36]. Approximately 15–20 % of severely injured motor vehicle occupants are trapped in their vehicle when emergency services arrive [37]. Therefore, on-scene treatment time usually takes longer in the majority of injured motor vehicle occupants than in injured pedestrians. Nevertheless, the expected mortality as calculated using the RISC-Score [19] and the observed mortality were higher in the pedestrian group. Utilizing the RISC-Score for the prediction of clinical outcome, several prognostic factors are assessed, rated and combined in a multivariate function. With respect to RISC-Score parameters, the injury itself and not the underlying collision mechanism mostly influence mortality. Age and severity of head injury exert the strongest impact on prognosis and mortality [19]. This corresponds well to the fact, that it is exactly these two factors, in which pedestrians and motor vehicle occupants differed the most in our observations.

Interestingly, the pedestrian group showed both a significantly higher rate of severe head injury as well as significantly less pre-hospital interventions, especially a lower pre-hospital intubation rate when compared to equally injured motor vehicle occupants. Despite the accordance of predicted and actual deaths in both groups, one has to conclude that the underestimation of pedestrian traffic collision victims during on-scene emergency treatment may contribute to their poor clinical prognosis. Same could be observed concerning operations in the hospital and prehospital sedation. Both groups show severe injuries making surgery necessary in over 80 % of cases. Pedestrians undergo operations in 81.2 % and motor vehicle occupants in 85.3 %. Our data can’t reveal an answer if this finding is caused by an underestimation or the circumstance that a part of the pedestrians die before entering the operation room. Concerning sedation 75 % of the pedestrian receive sedation whereat motor vehicle occupants are sedated in 84 %. As well in this finding more severe injuries contradict a less aggressive therapy regime. On the other hand a scoop and run regime of emergency teams for severely injured patients and short transport times could be an explanation for less pre-hospital treatment. Finally our data can’t reveal an exact explanation for these findings.

Our findings are important and reliable for several reasons. Most importantly, we included a very large number of patients, which makes the findings significant and representative. Moreover, software applications for plausibility were integrated in the data base, increasing the extent of representatively of the included patient population.

Nevertheless several limitations should be considered regarding this study. While approximately 90 % of all multiple trauma patients in Germany are estimated to be registered, participation at the TR-DGU is not obligatory. This might render the above mentioned findings somewhat less representative. Furthermore, all of the participating hospitals are active trauma centers. It is therefore conceivable that treatment-related findings in this study are overestimated when compared to non trauma centers. On the other side, this accounts for both the study and the control group, which compensates these aspects to some extent. Further relevant pitfalls of registry analyses in general are completeness of reported information, varying policies in medical care, the retrospective nature of this study design etc..

## Conclusion

Pedestrians are vulnerable road users with severe injuries and high subsequent mortality when involved in road traffic collisions. Emergency medicine team members should be sensitized to the “trigger term” pedestrian and anticipate the typical pedestrian motor vehicle collision victim as a very young or very old patient with significant head and lower extremity trauma and impaired level of consciousness and cardiovascular circulation. Further studies are needed in order to elucidate the incremental value of a lower threshold for pre-clinical intubation in this specific patient population versus a more Scoop and Run approach Finally, political and educational efforts must be made in terms of improved general awareness and traffic circumstances in order to achieve less collision related morbidity and mortality.

## Abbreviations

PMVC: Pedestrian motor vehicle collision
ISS: Injury severity score
AIS: Abbreviated injury scale
RISC-Score: Revised injury severity classification Score
SD: Standard deviation
PTT: Partial thromboplastin time
BE: Base excess
Hb: Hemoglobin
TR-DGU: TraumaRegister DGU®
